# Reliability, Validity, and Identification Ability of a Commercialized Waist-Attached Inertial Measurement Unit (IMU) Sensor-Based System in Fall Risk Assessment of Older People

**DOI:** 10.3390/bios13120998

**Published:** 2023-11-25

**Authors:** Ke-Jing Li, Nicky Lok-Yi Wong, Man-Ching Law, Freddy Man-Hin Lam, Hoi-Ching Wong, Tsz-On Chan, Kit-Naam Wong, Yong-Ping Zheng, Qi-Yao Huang, Arnold Yu-Lok Wong, Timothy Chi-Yui Kwok, Christina Zong-Hao Ma

**Affiliations:** 1 Department of Biomedical Engineering, The Hong Kong Polytechnic University, Hong Kong SAR, China; kejing.li@connect.polyu.hk (K.-J.L.); nickyly.wong@connect.polyu.hk (N.L.-Y.W.); tess.law@polyu.edu.hk (M.-C.L.); katherinehc.wong@polyu.edu.hk (H.-C.W.); tsz-on-jeffrey.chan@polyu.edu.hk (T.-O.C.); kn-jessica.wong@polyu.edu.hk (K.-N.W.); yongping.zheng@polyu.edu.hk (Y.-P.Z.); 2 Research Institute for Smart Ageing, The Hong Kong Polytechnic University, Hong Kong SAR, China; arnold.wong@polyu.edu.hk; 3 Jockey Club Smart Ageing Hub, The Hong Kong Polytechnic University, Hong Kong SAR, China; 4 Department of Rehabilitation Sciences, The Hong Kong Polytechnic University, Hong Kong SAR, China; freddy-mh.lam@polyu.edu.hk; 5 School of Fashion and Textiles, The Hong Kong Polytechnic University, Hong Kong SAR, China; qi-yao.huang@polyu.edu.hk; 6 Department of Medicine & Therapeutics, The Chinese University of Hong Kong, Hong Kong SAR, China; tkwok@cuhk.edu.hk

**Keywords:** fall risk assessment, wearable system, inertial measurement unit (IMU) sensor, the Mini-Balance Evaluation Systems Test (Mini-BESTest), community-dwelling older people

## Abstract

Falls are a prevalent cause of injury among older people. While some wearable inertial measurement unit (IMU) sensor-based systems have been widely investigated for fall risk assessment, their reliability, validity, and identification ability in community-dwelling older people remain unclear. Therefore, this study evaluated the performance of a commercially available IMU sensor-based fall risk assessment system among 20 community-dwelling older recurrent fallers (with a history of ≥2 falls in the past 12 months) and 20 community-dwelling older non-fallers (no history of falls in the past 12 months), together with applying the clinical scale of the Mini-Balance Evaluation Systems Test (Mini-BESTest). The results show that the IMU sensor-based system exhibited a significant moderate to excellent test–retest reliability (ICC = 0.838, *p* < 0.001), an acceptable level of internal consistency reliability (Spearman’s rho = 0.471, *p* = 0.002), an acceptable convergent validity (Cronbach’s α = 0.712), and an area under the curve (AUC) value of 0.590 for the IMU sensor-based receiver-operating characteristic (ROC) curve. The findings suggest that while the evaluated IMU sensor-based system exhibited good reliability and acceptable validity, it might not be able to fully identify the recurrent fallers and non-fallers in a community-dwelling older population. Further system optimization is still needed.

## 1. Introduction

Falls are a major health issue for older people worldwide, and about one-third of people aged older than 65 years fall each year [1]. Given the fact that China has the largest elderly population in the world, with 264.02 million older people (≥60 years old) [2], fall incidence and the subsequent injuries [3] in older people have placed a heavy burden on the health system. As the first step of a fall prevention program [4], fall risk assessment is an essential element to identify the people with a higher risk of falls and facilitate the provision of customized/individualized fall prevention interventions thereafter. However, existing fall risk assessment strategies vary a lot across different settings and populations [4]. A reliable and valid fall risk assessment strategy for community-dwelling older people is urgently needed.

Gait and balance disorders, as one of the most common causes of falls in older people [5], are significant predictors of falls [6]. Some clinical scales related to the evaluation of gait and balance disorders have been widely used as fall risk assessment tools, including the Berg Balance Scale (BBS), Timed Up and Go (TUG) test, Balance Evaluation Systems Test (BESTest), and Mini-Balance Evaluation Systems Test (Mini-BESTest). Among these clinical tests, the Mini-BESTest, a widely used comprehensive balance measurement scale consisting of 14 items [7], has been recommended as the most accurate tool for identifying the older people with a history of falls in the past 12 months [8]. Although all the above clinical scales were proven to exhibit good reliability and validity in fall risk assessment [9,10], they all require experienced professionals to conduct the assessment and are influenced by the assessors’ clinical experience, which potentially make them unsuitable for large-scale community-based fall risk screening.

Recently, some instrumented fall risk assessment tools have been developed and investigated to help provide more accurate, affordable, and easy-to-use services to communities [11,12]. Typical instrumented fall risk assessment tools include wearable sensing technology, floor-mounted force plates [13], electronic walkways [14], and posturography using motion capture systems [15]. Wearable sensing technology can be used to collect the users’ clinically relevant data accurately and provide an objective, continuous health monitoring service, by placing sensors into wearable objects or directly on the human body [16,17,18]. Compared to clinical scales and other instrumented assessment tools, wearable sensing technology has the advantages of being portable, unobtrusive, and inexpensive [19], which enables it to address the inevitable increasing need for daily/continuous fall risk monitoring and large-scale fall risk assessment in the current aging society [20].

The inertial measurement unit (IMU) is one commonly used wearable sensor that can be utilized in fall risk assessment. By incorporating an accelerometer, a gyroscope, and sometimes a magnetometer, the IMU can obtain the linear and angular acceleration, velocity, orientation, and gravitational force data of the target body segments accurately [21,22]. Studies have also suggested that IMU sensor-based systems can enhance the quantification/identification of standard clinical scales in fall risk assessment and prediction [23,24], and can be used in longitudinal monitoring protocols in daily activity [25]. However, so far, the reliability, validity, and identification ability of such IMU sensor-based systems in identifying community-dwelling older fallers and older non-fallers has remained inconclusive [26]. Therefore, before the implementation of IMU sensor-based technology in large-scale fall risk assessment programs for community-dwelling older people, the performance of IMU sensor-based systems in fall risk assessment needsto be carefully evaluated to ensure the promotion and execution of evidence-based practice.

Up to now, there has been a lack of evaluations of IMU sensor-based systems’ application in fall risk assessments for community-dwelling older people. This study bridged this research gap and evaluated the performance of a commercially available IMU sensor-based system from the aspects of reliability [27], validity [27], and identification ability with reference to the Mini-BESTest [28]. The findings of this study provide evidence on the current status of IMU sensor-based systems in assessing fall risks of community-dwelling older people, and they are expected to inspire future development and optimization of such instrumented fall risk assessment tools.

## 2. Methods

### 2.1. Participants

This study was conducted at the Jockey Club Smart Aging Hub of the Hong Kong Polytechnic University. A total of 40 participants, including 20 community-dwelling older recurrent fallers (≥65 years old and had a history of 2 or more falls in the past 12 months) [29] and 20 community-dwelling older non-fallers (≥65 years old and with no history of falls in the past 12 months), were recruited through the convenience sampling via the Jockey Club Smart Aging Hub. This sample size of 40 participants yielded a statistical power of 0.809, suitable for the case–control study design, with an effect size of 0.92 based on the published literature [30] and a significant level of 0.05. None of the participants had previously participated in similar trials or studies involving wearable systems. Participants were excluded if they had any active medical conditions or active inflammatory/pathologic changes in lower-extremity joints within six months prior to the experiment. All data collected were kept confidential and identifiable only through codes known to the researchers. This study adhered to the principles of the Declaration of Helsinki and received approval from the Institutional Review Board of the Hong Kong Polytechnic University (Reference number: HSEARS20211116005). All participants provided written informed consent before the experiments.

### 2.2. Equipment

**Figure 1:**
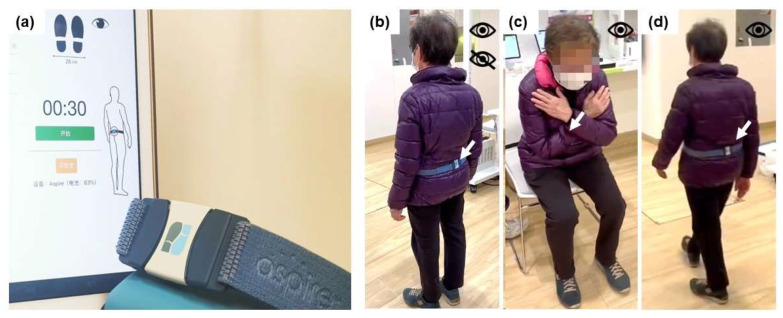
(**a**) The validated IMU sensor-based system in this study. (**b**–**d**) Illustrations of the system attachment during assessment on one participant as an example: (**b**) stand still with eyes open (sub-task 1); stand still with eyes closed (sub-task 2); (**c**) sit and stand repeatedly with eyes open (sub-task 3); and (**d**) walk at normal speed with eyes open (sub-task 4).

A commercially available 9-axis inertial measurement unit (IMU)-based system (Booguu Aspire^TM^ Fall Risk Management System, Booguu Company Limited, Hong Kong SAR, China) was used and evaluated in this study (Figure 1a). This system, as described in the user manual and on the company website [31], was developed to offer a simple, fast, and accurate fall risk assessment for community-dwelling older people. Specifically, the system includes a waistbelt with an embedded 6-axis accelerometer/gyroscope and an embedded 3-axis magnetometer for data detection, a tablet application for user operation, and an online database for data processing and storage (Table 1). To conduct the fall risk assessments, users were instructed to complete a 5-min task [32]. Throughout the assessments, data captured by the waistbelt were uploaded to the online database for further data processing. The whole assessment procedure could be monitored and controlled via the tablet application, with a comprehensive report containing the assessment results generated after each assessment (Supplementary Material: Fall Risk Assessment Report).

To apply the system for fall risk assessment, the waistbelt was placed tightly against the end user’s lower back using an elastic strap. The 5-minute task included four sub-tasks [32]: (1) Postural stability I (Figure 1b): stand still for 30 s in a 28 cm × 28 cm space with eyes open, to assess the health condition of the sensory system and nervous system under eyes-open conditions; (2) Postural stability II (Figure 1b): stand still for 30 s in a 28 cm × 28 cm space with eyes closed, to assess the health condition of the sensory system and nervous system under eyes-closed conditions; (3) Dynamic movement (Figure 1c): cross hands and rest on the chest, and thensit and stand repeatedly at the fastest pace for 15 seconds, to assess the functional capacity of the lower limbs; and (4) Gait (Figure 1d): walk at a normal speed for about 10 meters, to assess an individual’s walking pattern. The final total assessment result was represented by scores ranging from 1 to 10. Additionally, after each assessment, the system generated separate results for each of the four sub-tasks, which were also represented by scores ranging from 1 to 10. These results were obtained through a patented categorization algorithm and the company’s own database [33,34,35]. As specified in the user manual, total scores falling within the range of 1 to 3, 4 to 7, and 8 to 10 indicated the high, medium, and low risks of falls, respectively.

### 2.3. Experimental Protocol

Experiments were conducted in an open room at the Jockey Club Smart Aging Hub, the Hong Kong Polytechnic University, where participants had a spacious and quiet environment in which to undergo the assessments without interruptions. All participants received guidance and protection from the same experienced researcher to keep consistency in data collection and interpretation. The entire experiment was completed within a one-hour timeframe on the same day for each participant. The experiment consisted of three parts: demographic data collection, instrumented fall risk assessment, and clinical fall risk assessment. The demographic data collection was completed by documenting participants’ self-reported age, gender, medication history, and fall history, followed by onsite measurements of weight and height. The instrumented fall risk assessment was completed by conducting three assessments using the IMU sensor-based system. The clinical fall risk assessment was completed by conducting one assessment using the Mini-BESTest. The sequence of the three IMU sensor-based assessments and the Mini-BESTest was randomized for each participant to minimize potential order effects. A five-minute rest was provided between each assessment to mitigate fatigue and minimize possible learning effects.

### 2.4. Data and Statistical Analysis

Overall, the instrumented fall risk assessment results were represented by the total and sub-tasks score generated from the IMU sensor-based system, ranging from 0 to 10 [32]. The clinical fall risk assessment results were represented by the scores generated from the Mini-BESTest, ranging from 0 to 28 [36]. Statistical analysis was conducted using the Statistical Package for Social Sciences (SPSS, version 26.0, IBM Corporation, Armonk, NY, USA). The detailed data and statistical analysis can be divided into four parts as follows:

#### 2.4.1. Reliability Evaluation of the IMU Sensor-Based System

The test–retest reliability of the IMU sensor-based system was assessed using the total and sub-task scores obtained from the three IMU sensor-based assessments. A single-measurement, absolute-agreement, 2-way random-effects model of the intra-class coefficient (ICC) [37] was used to examine the test–retest reliability of the IMU sensor-based system.

The internal consistency reliability of the IMU sensor-based system was assessed using the averaged total and sub-task scores obtained from the three IMU sensor-based assessments. Cronbach’s coefficient α [38] was used to examine the internal consistency reliability of the IMU sensor-based system.

#### 2.4.2. Validity Evaluation of the IMU Sensor-Based System

The convergent validity of the IMU sensor-based system was assessed by calculating the correlation between the averaged total scores obtained from the three IMU sensor-based assessments and the scores obtained from the Mini-BESTest. Spearman’s rank correlation coefficient was used to examine the convergent validity of the IMU sensor-based system [39,40].

#### 2.4.3. Identification Ability Evaluation of the IMU Sensor-Based System

The fall risk identification ability of the IMU sensor-based system was assessed by using the receiver-operating characteristic (ROC) of the averaged total scores obtained from the three IMU sensor-based assessments [41]. The sensitivity and specificity of the IMU sensor-based system in fall risk identification were further analyzed based on the cut-off scores and the participants’ groups (recurrent fallers and non-fallers). Three cut-off scores were used for analysis: one based on the ROC result of this study, and the other two based on the system’s recommended thresholds for identifying fall risks in older people (3.5 and 7.5 points [32]).

#### 2.4.4. Identification Ability Evaluation of the Mini-BESTest

Similar to the IMU sensor-based system, the fall risk identification ability of the Mini-BESTest was also assessed in this study, by analyzing the receiver-operating characteristic (ROC) of the scores obtained from the Mini-BESTest [41]. The sensitivity and specificity of the Mini-BESTest in fall risk identification were analyzed based on the cut-off scores and the participants’ groups (recurrent fallers and non-fallers). Three cut-off scores were used for analysis: one based on the ROC result of this study, and the other two based on recommendations from two previous studies for identifying fall risks in older people using the Mini-BESTest (16 points [8] and 19.5 points [42]).

## 3. Results

### 3.1. Demographic Data

As shown in Table 2, a total of forty community-dwelling older people (age: 70.4 ± 5.9 years; gender: 18 males and 22 females; height: 160.1 ± 9.6 cm; weight: 60.4 ± 11.3 kg; BMI: 23.5 ± 3.7 kg/m^2^) participated in this study, including twenty recurrent fallers and twenty non-fallers. No significant difference was observed between the recurrent faller and non-faller groups in age (*p* = 0.605), height (*p* = 0.351), weight (*p* = 0.631), or BMI (*p* = 0.083). No falls or any other adverse events occurred during the experiments.

### 3.2. Reliability of the IMU Sensor-Based System

Regarding the test–retest reliability, the results are presented in Table 3. Specifically, good to excellent test–retest reliability was observed in the total scores (ICC = 0.838, *p* < 0.001), moderate to good test–retest reliability was observed in the first (ICC = 0.717, *p* < 0.001) and the second (ICC = 0.698, *p* < 0.001) sub-task scores, poor to good test–retest reliability was observed in the third sub-task scores (ICC = 0.653, *p* < 0.001), and good to excellent test–retest reliability was observed in the fourth sub-task scores (ICC = 0.843, *p* < 0.001).

Regarding the internal consistency reliability of the IMU sensor-based system, an acceptable level of internal consistency reliability was observed (containing four sub-tasks, Cronbach’s α = 0.712).

### 3.3. Validity of the IMU Sensor-Based System

**Figure 2:**
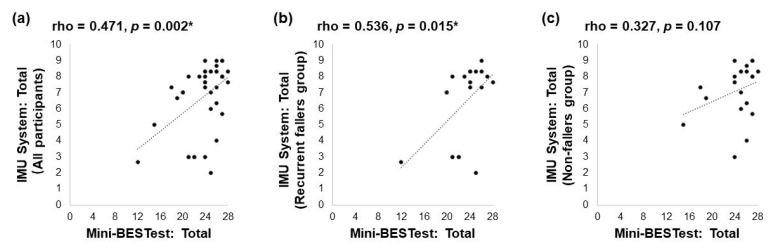
Correlation between the scores of Mini-BESTest and the scores of the IMU sensor-based system in (**a**) all participants, (**b**) recurrent fallers only, and (**c**) non-fallers only (* *p* < 0.05).

As shown in Figure 2, there were also significant moderate positive correlations [43] between the scores of the Mini-BESTest and those of the IMU sensor-based system in all participants (Spearman’s rho = 0.471, *p* = 0.002, Figure 2a) and in the recurrent faller group (Spearman’s rho = 0.536, *p* = 0.015, Figure 2b). No significant correlation was observed in the non-faller group (Spearman’s rho = 0.327, *p* = 0.107, Figure 2c).

**Figure 3:**
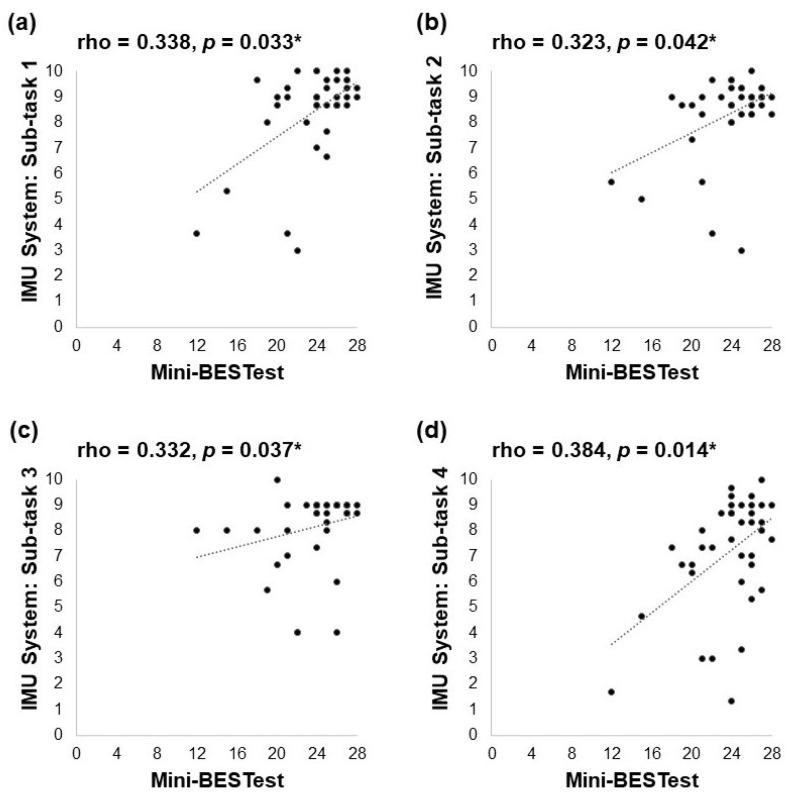
Correlation between the scores of Mini-BESTest and the sub-task scores of the IMU sensor-based system for (**a**) sub-task 1; (**b**) sub-task 2; (**c**) sub-task 3; and (**d**) sub-task 4 (* *p* < 0.05).

The sub-task score analysis of the IMU sensor-based system is shown in Figure 3. For all participants, there were significant though weak positive correlations between the scores of the Mini-BESTest and those of the first sub-task (Spearman’s rho = 0.338, *p* = 0.033, Figure 3a), the second sub-task (Spearman’s rho = 0.323, *p* = 0.042, Figure 3b), the third subtask (Spearman’s rho = 0.332, *p* = 0.037, Figure 3c), and the fourth sub-task (Spearman’s rho = 0.384, *p* = 0.014, Figure 3d) of the IMU sensor-based system.

### 3.4. Fall Risk Identification Ability of the IMU Sensor-Based System

**Figure 4:**
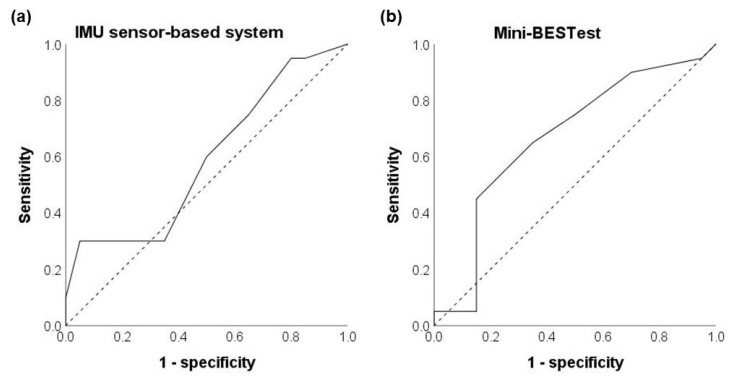
The ROC curves of the (**a**) IMU sensor-based system and (**b**) Mini-BESTest. Dotted lines represent random classifiers.

As shown in Figure 4a, the area under the curve (AUC) of the IMU sensor-based system was 0.590 (with 95% confidence interval = 0.411–0.769, *p* = 0.330, Figure 4a). According to the IMU sensor-based system’s ROC curve, the best sensitivity and specificity values were achieved with a cut-off score of 3.5/10, which aligned with one of the recommended cut-off scores by the company. Using this cut-off score, the IMU sensor-based system demonstrated a sensitivity of 30% in correctly identifying recurrent fallers and a specificity of 95% in correctly identifying non-fallers.

When the cut-off score of 7.5/10 [32] was used, the IMU sensor-based system demonstrated a sensitivity of 50% in correctly identifying recurrent fallers and a specificity of 55% in correctly identifying non-fallers.

### 3.5. Fall Risk Identification Ability of the Mini-BESTest

As shown in Figure 4b, the area under the curve (AUC) of the Mini-BESTest was 0.668 (with 95% confidence interval = 0.494–0.841, *p* = 0.070, Figure 4b). According to the Mini-BESTest’s ROC curve, the best sensitivity and specificity values were achieved with a cut-off score of 24.5/28. Using this cut-off score, the Mini-BESTest demonstrated a sensitivity of 65% in correctly identifying recurrent fallers and a specificity of 65% in correctly identifying non-fallers.

When the cut-off scores of 16/28 [8] and 19.5/28 [42] were used, the Mini-BESTest demonstrated sensitivities of 5% and 5% and specificities of 95% and 85%, respectively.

## 4. Discussion

The performance of a commercialized waist-attached inertial measurement unit (IMU) sensor-based system in fall risk assessment of community-dwelling older people was evaluated in this study. Generally, the findings of this study indicate that: (1) the IMU sensor-based system exhibited good reliability, acceptable validity, and poor identification ability; and (2) the Mini-BESTest exhibited inconclusive identification ability in the fall risk assessments of community-dwelling older people. More details are provided in the subsequent sections.

### 4.1. Good Reliability of the IMU Sensor-Based System

The significant moderate to excellent test–retest reliability and the acceptable level of internal consistency reliability of the IMU sensor-based system generally indicated that such a system possesses good reliability in fall risk assessment of community-dwelling older people. This aligns with previous research that corroborated the reliable performance of IMU sensors in detecting a range of balance-related biomechanical signals [26,44,45], as well as in fall risk assessments of patients with multiple sclerosis [46] and stroke [47]. It is also noteworthy that the good test–retest reliability of the average total scores of the IMU sensor-based system (ICC = 0.838) is comparable to that of the commonly used clinical balance tests, including the BBS, the BESTest, the Mini-BESTest, and the Brief-BESTest (0.886 ≤ ICC ≤ 0.945) in fall risk assessments of older people [9]. These results lend support to the notion that IMU sensors can yield considerably reliable results for fall risk assessment of community-dwelling older people, especially when compared with the existing, widely applied clinical balance assessments.

### 4.2. Acceptable Validity of the IMU Sensor-Based System

The significant moderate positive correlation between the averaged total scores of the IMU sensor-based system and the scores of the Mini-BESTest suggests that the IMU sensor-based system exhibited acceptable convergent validity in fall risk assessment of community-dwelling older people. It is also encouraging to note the significant though weak positive correlations between the four sub-task scores of the IMU sensor-based system and the scores of the Mini-BESTest. The Mini-BESTest is a widely utilized comprehensive balance measurement scale comprising 14 items [7], and it includes four balance-related subscales: anticipatory, reactive postural control, sensory orientation, and dynamic change [48]. The observed acceptable convergent validity indicates that the IMU sensor-based system aligns with the Mini-BESTest, suggesting that the IMU sensor-based system could potentially serve as an efficient adjunct or supplement to the Mini-BESTest in fall risk assessment of older people.

When we consider assessments besides the Mini-BESTest, the IMU sensor-based technology has already been proven to be effective in enhancing other clinical balance assessment scales in previous studies, such as the Timed Up and Go (TUG) test and the Berg Balance Scale (BBS), by providing automated kinematic data assessments [23,24,49,50]. Given that most clinical scales used for fall risk assessments of older people need to be applied by professional experts, which requires substantial health resources and manpower, it has been challenging to provide widespread, long-term, cross-institutional fall-risk screening services for older people in the community. In light of the positive outcomes of this study, the IMU-related technology may serve as a surrogate or supplement of clinical scales in large-scale fall risk assessment [51,52,53] of community-dwelling older people in future clinical practice.

### 4.3. Poor Identification Ability of the IMU Sensor-Based System

This study observed an AUC value (AUC value = 0.590, *p* = 0.330) in the IMU sensor-based system ROC curve that was not meaningfully greater than chance. This suggests that the IMU sensor-based system may not be able to effectively distinguish between recurrent fallers and non-fallers among community-dwelling older people. The identification ability of an IMU sensor-based system is determined by multiple factors, and such negative results could be explained by the following aspects.

The placement of the IMU sensor and the detected parameters need careful consideration and evaluation. While most studies place the IMU sensor close to the center of the body for body orientation detection [20], other commonly used strategies include placing the IMU sensor on a lower limb [54], the sternum [55], or the head [56] for the detection of gait parameters, trunk acceleration, and head stabilization, respectively. It can generally be concluded that placing IMU sensors on different body parts could be a good means of comprehensive parameter detection and analysis. For example, Liu et al. [51] used a single IMU attached to different body parts for the detection of different parameters, suggesting that the combination of different parameters performed better in fall risk identification compared to individual parameters. However, there is currently a lack of guidelines on IMU sensor placement and parameter detection specifically tailored to fall risk assessment, especially for community-dwelling older people. Future protocol developments and investigations are, therefore, necessary.

The classification algorithms and scoring criteria need optimization and further investigation. Given that a fall is a multifactorial event, different fall risk classification algorithms and scoring criteria are needed for assessing different parameters and for use in older people with different health conditions. Previous studies have demonstrated that the integration of machine learning models [50] and deep learning approaches [57,58] with IMU sensor-based technology can improve fall risk identification accuracy. However, these studies exhibit considerable variation in terms of the population, the utilized algorithms, and the scoring criteria. Further investigations are needed, not only on the improvement of fall risk classification accuracy but also on the establishment of correspondence between the scoring and its clinical implications.

The sensor type and number also require further evaluation and optimization. For example, Qiu et al. [59] reported that five IMUs worn on the lower back and lower limbs could accurately assess fall risk in older people. The force sensor, another type of commonly used wearable motion sensor, has been widely used together with IMU sensors to develop fall risk assessment devices/systems [54,60]. Such positive findings indicate that integrating multiple IMUs with other types of wearable motion sensors could be helpful for future studies to provide more comprehensive assessments and address the current unsatisfactory results of using the single IMU sensor-based system, as identified in this study.

Unfortunately, the details of the IMU sensor-based system, including the raw data and parameters detected, background algorithms, scoring criteria, and other technical details (e.g., sampling frequency, ranges), were not disclosed by the company. While the user manual recommended tightening the IMU sensor at the lower back, the reason for this placement was not provided. Disclosing more technical details would not only enable the investigation and explanation of the current insignificant findings but also facilitate the optimization of such commercial IMU sensor-based systems. This would ultimately lead to more accurate and validated assessment results in identifying the risk of falls among community-dwelling older people in the future.

### 4.4. Inconclusive Identification Ability of the Mini-BESTest

This study observed an AUC value of 0.668 (*p* = 0.070) with marginal significance in the Mini-BESTest ROC curve. Given that ROC curves with an AUC ≤ 0.75 are not considered clinically useful/significant, the ability of the Mini-BESTest to distinguish between recurrent fallers and non-fallers among community-dwelling older people remains uncertain. This result aligns with a previous study, which also reported that the Mini-BESTest had poor fall risk identification ability (AUC = 0.54, 58 older people, mean age = 78 years) in older adults with self-reported balance problems [61]. It should be noted that as a commonly used clinical scale, the Mini-BESTest has been recommended as a stable and reliable tool for fall risk detection in older people [61] and has been shown to be more accurate than the BESTest, TUG, and BBS tests in identifying older people’s fall history [8]. Previous studies investigating the Mini-BESTest’s accuracy in identifying older people with a history of falls in the past 12 months, have found positive results and reported the cut-off scores to be 16/28 (AUC = 0.84, 200 older people, mean age = 70) [8] and 19.5/20 (AUC = 0.76, 122 older people, mean age = 76) [42], respectively. Despite these inconsistent findings above, one study involving 264 older people found that Mini-BESTest performed well in fall risk prediction for older adults, with different cut-off scores corresponding to different age groups [62], which suggested that recruiting more participants, preferably with a different grouping method and more specific subject inclusion/exclusion criteria, could be potential promising ways to conduct future in-depth investigations on the Mini-BESTest’s fall risk identification ability in older people.

### 4.5. Limitations

There are several limitations of this study. Firstly, this study had a relatively small sample size, and the participants were categorized as recurrent fallers or non-fallers based on their self-reported fall history without multi-labeling. During the data collection phase, it was observed that some participants categorized as “recurrent fallers” were highly active and frequently engaged in outdoor activities such as hiking or street running, which increased their exposure and likelihood of falling. Conversely, some participants categorized as “non-fallers” exhibited poor balance ability (as evidenced by the Mini-BESTest score) and tended to live a sedentary life, leading to less exposure and likelihood of falling. Given that the fall risk increases for both the highly inactive and the highly active older people as compared to those who are moderately active [63], the current self-reported data collection strategy cannot fully reflect the participants’ true statuses and is susceptible to memory bias. Thus, implementing regular prospective follow-up interviews and standardized questionnaires, such as the International Physical Activity Questionnaire modified for the elderly (IPAQ-E) [64], to collect fall events and physical activity levels could provide a more reliable reference for the grouping method. Furthermore, considering that factors such as age, gender [65], and co-morbidities/diseases such as poliomyelitis [66] and diabetes [67] can significantly affect the balance performance and the occurrence of falls, future studies with larger sample sizes and more diverse populations should be conducted to address this limitation.

Secondly, there might be some learning effects that existed across different trials in the current experimental protocol. Despite incorporating a 5-minute recovery time between each trial to mitigate potential fatigue and interactions, the learning effect may still have existed. To achieve a more accurate evaluation of the test–retest reliability of the system, conducting reliability assessments on different days would offer a superior assessment protocol.

Thirdly, this study has evaluated the performance of the IMU sensor-based system and compared it only to the Mini-BESTest. It should be noted that different outcomes may arise when comparing the system with other commonly employed clinical balance assessments. While the Mini-BESTest has been suggested to be the most accurate tool in fall risk identification for older people among most of the clinical balance scales [8], researchers also highlighted the absence of a universally recommended tool (or “gold standard”) for fall risk assessments [68]. By undertaking comparisons between the IMU sensor-based system and other clinical scales, a more comprehensive understanding of the evaluation results and their interpretations can be attained.

Finally, this study adopted a retrospective study design without prospective follow-ups. Incorporating a prospective study design in fall risk assessment studies would not only support unambiguous data collection concerning fall events but also enable the investigation of the system’s fall prediction abilities. Furthermore, future research should address the long-term user compliance and solicit feedback regarding the system’s acceptability. This research should involve both the end users, such as older people and individuals with disabilities, and the assessors, including clinicians and rehabilitation therapists. Conducting such studies will further support the community-based implementation and integration of the system into clinical practice.

## 5. Conclusions

This study has bridged a research gap and provided insights into the application of IMU sensor-based systems in fall risk assessments of community-dwelling older people. Specifically, the study has evaluated the performance of a commercially available IMU sensor-based system in comparison with the Mini-BESTest. Good reliability, acceptable validity, but poor identification ability have been identified for the commercialized IMU sensor-based system in assessing the fall risk among community-dwelling older people.

Despite the relatively poor performance of the evaluated system in identifying fall risks, the potential of IMU sensor-based technology in facilitating long-term fall risk screening among community-dwelling older people cannot be denied. This is particularly true when considering its compact size, low cost, good reliability, acceptable validity, and the generally accurate analysis of motion parameters. It is recommended that healthcare practitioners can use the IMU sensor-based system as a valuable reference/adjunct/supplement due to its objective and accurate motion parameter detection and analysis, but we advise taking a cautious approach when providing diagnoses or clinical suggestions solely based on the system’s results. Future optimization of the IMU sensor-based systems remains necessary, not only for the improvement of fall risk identification accuracy but also for the exploration of the relevant clinical implications for community-dwelling older people.

## Figures and Tables

**Table 1:** Technical specifications of the IMU sensor-based system.

Items	Specifications
Dimensions	72.6 mm × 40.5 mm × 12.6 mm
Weight	32 g
Processor	ARM^®^ Cortex™-M4F 32-bit processor
Accelerometer/Gyroscope	Bosch ^®^ 6-axis accelerometer/gyroscope
Magnetometer	Bosch ^®^ 3-axis magnetometer
Inertial sensor platform	Bosch ^®^ 9-axis sensor fusion
Battery	Rechargeable lithium ion/polymer battery

**Table 2:** Participants’ demographic data (mean ± SD).

	Females (*n* = 22)	Males (*n* = 18)	Recurrent Fallers (*n* = 20)	Non-Fallers (*n* = 20)	All Participants (*n* = 40)
Age (year)	70.6 ± 6.8	70.1 ± 4.7	70.8 ± 6.7	70.0 ± 5.2	70.4 ± 5.9
Height (cm)	154.2 ± 7.5	167.3 ± 6.4	158.6 ± 10.5	161.5 ± 8.5	160.1 ± 9.6
Weight (kg)	54.2 ± 8.8	68.0 ± 9.4	61.3 ± 10.3	59.6 ± 12.4	60.4 ± 11.3
BMI (kg/m²)	22.9 ± 4.0	24.3 ± 3.2	24.3 ± 3.0	22.8 ± 4.2	23.5 ± 3.7
Total score of the IMU sensor-based system	6.7 ± 2.0	6.9 ± 2.4	6.4 ± 2.5	7.2 ± 1.7	6.8 ± 2.1
Total score of the Mini-BESTest	22.8 ± 3.6	25.0 ± 3.0	23.1 ± 3.5	24.5 ± 3.4	23.8 ± 3.5

**Table 3:** The intraclass correlation coefficient (ICC) values of the total and sub-task scores generated by the IMU sensor-based system during three assessments.

Score	ICC Value	95% Confidence Interval		*p*-Value
		Lower Bound	Upper Bound	
Total	0.838	0.745	0.904	<0.001
1st task (postural stability with eyes open)	0.717	0.576	0.827	<0.001
2nd task (postural stability with eyes closed)	0.698	0.553	0.814	<0.001
3rd task (dynamic movement and gait I)	0.653	0.495	0.783	<0.001
4th task (dynamic movement and gait II)	0.843	0.753	0.907	<0.001

## Data Availability

Data are contained within the article.

## Supplementary Materials

The following supporting information can be downloaded at: https://www.mdpi.com/article/10.3390/bios13120998/s1. An example of the report generated by the IMU sensor-based system. (Note: The original report was in Chinese, and was translated into English by the authors. The original Chinese-version report is provided in the later pages of the translated English-version report. In case of any ambiguity, precedence should be given to the Chinese version).
