# Supplementary material for: Reliability, Validity, and Identification Ability of a Commercialized Waist-Attached Inertial Measurement Unit (IMU) Sensor-Based System in Fall Risk Assessment of Older People
Source: Biosensors (Basel). 2023 Nov 25;13(12):998. doi: 10.3390/bios13120998 (PMC10742152; doi:10.3390/bios13120998)
Supplement: Supplementary file 1 [file biosensors-13-00998-s001.zip › biosensors-2634641-supplementary.pdf]

Note:
The original report was in Chinese, and was translated into English by the authors. The original Chinese-version report is provided in the subsequent pages of the translated English-version report. In case of any ambiguity, precedence should be given to the Chinese version.

Name	Gender	Age	Height	Weight	Date

Fall Risk Assessment Report

Fall Risk (based on body movement)	High			Medium				Low		
	1	2	**3**	4	5	6	7	8	9	10

Suggestions

1. Consult your doctor about the use of medication and its side effects.
2. Participate in fall prevention programs to improve mobility, lower limb strength, and balance.
3. Managing vision, orthostatic hypotension, and foot problems.
4. It is recommended to improve home safety through a home assessment.

Body motion assessment	Poor	Moderate	Good
Gait - natural walking		7	
Assessing an individual's walking pattern to identify fall risk indicators, including variability, strength, and neuromuscular control. Score: 1 - 10 points (0 = unable to complete safely).			
Postural stability - eyes open		4	
Postural stability - eyes closed		6	
Assessing the health condition of the sensory system and nervous system that contribute to maintaining balance. Score: 1 - 10 points (0 = unable to complete safely)			
Dynamic movement - sit and stand	3		
Assessing the functional capacity of the lower limbs to support movements from sitting to standing and returning to sitting. Score: 1 - 10 points (0 = unable to complete safely).			

Self-reported physical condition	
Have you experienced any falls in the past 6 months?	No
Do you feel dizzy when getting up quickly?	Yes
How is your vision under indoor lighting conditions?	Good
How many prescription medications do you take daily?	<4

Copyright 2023, Booguu Co. Ltd. info@booguu.bio +852 3590 2685

Note:
The original report was in Chinese, and was translated into English by the authors. The original Chinese-version report is provided in the subsequent pages of the translated English-version report. In case of any ambiguity, precedence should be given to the Chinese version.

Name	Gender	Age	Height	Weight	Date

About the Fall Risk Assessment Report

Strength, balance, and coordination are key for regaining control of the body during slips or trips. Walking, balance, and the "sit-to-stand test" assess these important factors. If the score in any of these tests does not exceed 3 points, it may indicate the need for further medical follow-up.

It is advisable to incorporate strength, balance, and flexibility training into daily activities to improve or maintain scores. Before starting any new exercise program, it is important to consult with a doctor.

Walking performance

Walking performance (gait) testing can measure the strength, coordination, and stability of lower body. Individuals with a healthy gait exhibit at least a minimum walking speed and consistent step-to-step coordination. Possible reasons for a lower gait score include slow speed, shuffling gait, excessive and unnecessary lateral swaying, or inconsistent step-to-step coordination.

Balance in standing

Balance testing can measure the subtle movements of the body during standing. Standing involves three systems: vision, the inner ear (also known as the vestibular system), and proprioception. When one or more of these systems undergo changes, balance may be affected.

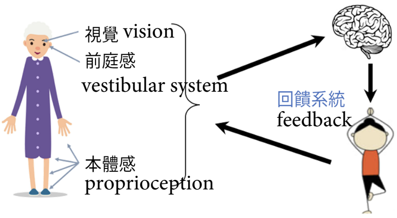

A low balance score indicates frequent significant adjustments of the body while standing. If there is a noticeable difference in balance when the eyes are open versus closed, it suggests a high reliance on vision for maintaining balance, and there may be potential issues with the proprioceptive system responsible for self-motion perception.

"Five Times Sit-to-Stand" test

The "Sit-to-Stand" test measures lower body strength and stability. Healthy individuals can complete 5 cycles of the movement with stable and sufficient upward force, and the time taken falls within the reference range corresponding to their age.

Age	Reference time (s)
≤ 69	11.4
70 - 79	12.6
≥ 80	14.8

A lower score may indicate insufficient lower body strength or unstable body movements.

Note:
The original report was in Chinese, and was translated into English by the authors. The original Chinese-version report is provided in the subsequent pages of the translated English-version report. In case of any ambiguity, precedence should be given to the Chinese version.

Name	Gender	Age	Height	Weight	Date
[redacted]					

Gait assessment (with shoes)

Forward acceleration

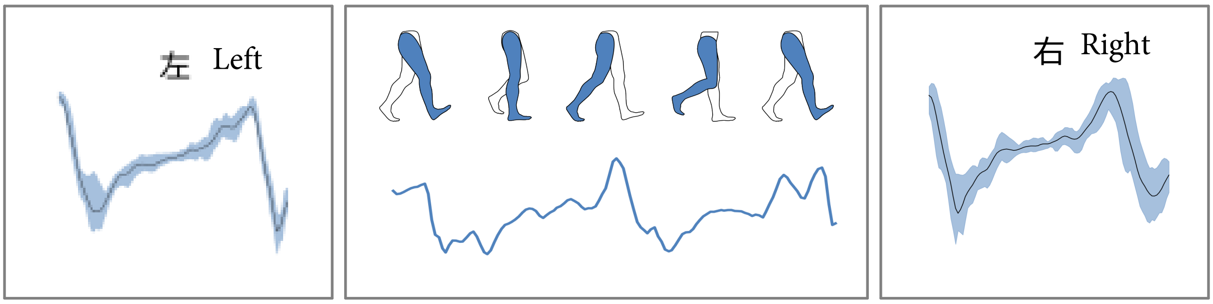

Fall risk of gait

7/10 Moderate

Assessing walking patterns includes evaluating the variability, intensity, and neuromuscular control of time and movement.

Gait risk factors	Scores	
Performance of gait		
Energy expenditure per step	9	Good
Vertical displacement of movement	7	Moderate
Hip/pelvic stability	8	Good
Step-to-step variability	8	Good

More info			Reference range
Step frequency (steps/minute)	113		> 96
Temporal asymmetry (%)	5.1		< 6.9
Landing force asymmetry (%)	1.4		< 14.5
Average landing force (× body weight)	1.45	1.47	1.44 - 2.05

Copyright 2023, Booguu Co. Ltd. info@booguu.bio +852 3590 2685

Note:
The original report was in Chinese, and was translated into English by the authors. The original Chinese-version report is provided in the subsequent pages of the translated English-version report. In case of any ambiguity, precedence should be given to the Chinese version.

Name	Gender	Age	Height	Weight	Date

Balance (static stability) assessment

Body sway trajectory & length

Open eyes

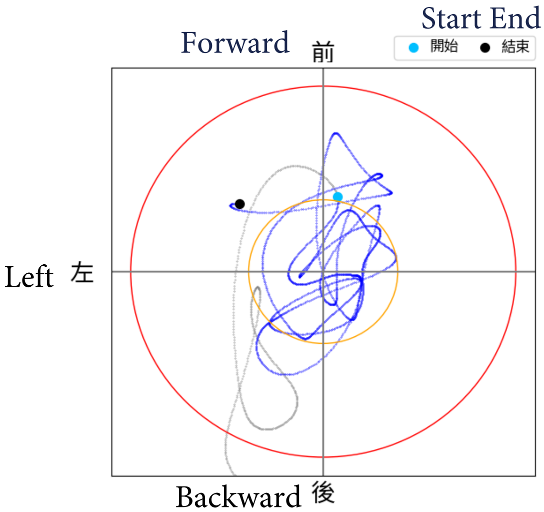

Close eyes

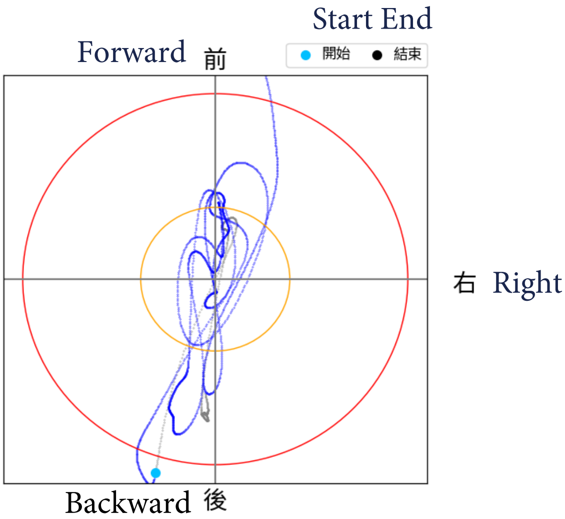

		Reference	Fall risk
Swing length when eyes open	29.3cm	< 33.4cm	4/10
Swing length when eyes closed	29.0cm	< 38.0cm	6/10
Romberg test			Negative

Swing length is the sum of the length of the body's movement trajectory. A shorter swing length indicates better stability.
The Romberg test compares balance results between open eyes and closed eyes. A positive result in the Romberg test may indicate a reliance on vision for balance or inadequate vestibular and proprioceptive senses.

Copyright 2023, Booguu Co. Ltd. info@booguu.bio +852 3590 2685

aspire™

Note:
The original report was in Chinese, and was translated into English by the authors. The original Chinese-version report is provided in the subsequent pages of the translated English-version report. In case of any ambiguity, precedence should be given to the Chinese version.

Name	Gender	Age	Height	Weight	Date

Sit-to-Stand assessment

Five Times Sit-to-Stand

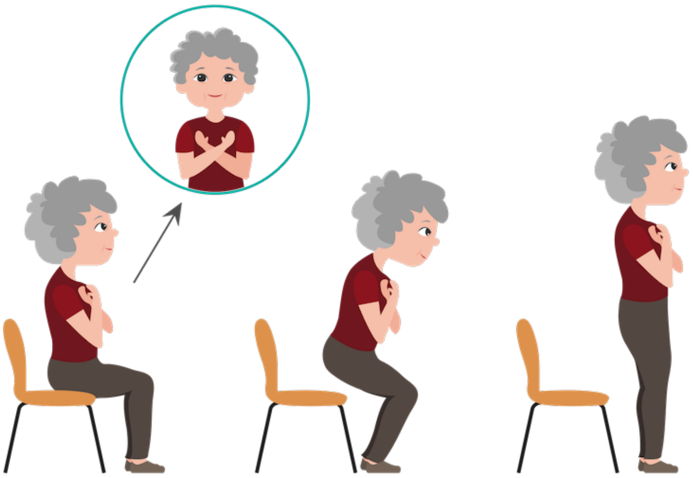

Fall risk of Sit-to-Stand

3/10 Poor

Assessing functional lower limb strength and the ability to transition from a seated position to standing.

		Reference
Have completed 5 repitetions	No	
Number of completed repetitions	4	
Time spent to complete 5 repetitions (s)	N/A	< 11.40
Time spent to the last stand (s)	12.27	
Standing power (N)*	1.28	0.71 - 4.14
Functional efficiency	8	> 3
Decent control	No	

* multiply body weight (kg)

Sit-to-stand measures the vertical acceleration during the transition from a seated to a standing position and assesses the strength required for this movement. The higher the power output, the stronger the lower limb muscles. Functional efficiency measures the quality of movement transitions from sitting to standing and from standing to sitting. Inability to control the descent may indicate weakness, pain, restricted joint mobility, or decreased attention.

Copyright 2023, Booguu Co. Ltd. info@booguu.bio +852 3590 2685

Note:
The original report was in Chinese, and was translated into English by the authors. The original Chinese-version report is provided in the subsequent pages of the translated English-version report. In case of any ambiguity, precedence should be given to the Chinese version.

Name	Gender	Age	Height	Weight	Date

Beware of traps in the environment:
https://www.elderly.gov.hk/tc_chi/healthy_ageing/home_safety/falls.html

Be a tumbler:
https://www.elderly.gov.hk/tc_chi/books/files/fall_prevention/Fall_Prevention_booklet.pdf

For explanations of the relevant parameters, please refer to the following document:
https://aspiremotion.com/rg.zh-hant.pdf

The information contained in this report is of a general nature and does not substitute for advice provided by your doctor or other healthcare professionals. Balance and gait scores are for reference purposes only and are not medical diagnoses or assessments.
These statements are not recommendations for treating any specific disease or health-related condition. If you suspect you have a medical condition or health issue, you should immediately contact a healthcare professional. Before starting any balance training, rehabilitation program, dietary adjustments, health exercises, or initiating any supplementation or medication treatment plan, please consult with a healthcare professional.

Copyright 2023, Booguu Co. Ltd. info@booguu.bio +852 3590 2685

名字	性別	年齡	身高	體重	日期

跌倒風險評估

整體跌倒風險（基於身體動作）	高			中				低		
	1	2	**3**	4	5	6	7	8	9	10

建議

1. 向您的醫生咨詢藥物的使用及其副作用。
2. 參加防跌管理班去改善活動能力，下肢力量與平衡能力。
3. 處理視力，姿勢性低血壓及足部問題。
4. 建議透過家居評估改善家居安全。

身體動作評估	較差	適中	良好
**步態**—行走		7	

評估個人行走的方式，以識別跌倒風險指標，包括變化性、力量和神經肌肉控制。 分數：1 – 10分 (0 = 不能安全完成)

身體動作評估	較差	適中	良好
**靜態控制**—睜眼		4	
**靜態控制**—閉眼		6	

測量有助於維持平衡的感覺系統和神經系統的健康狀況。 分數：1 – 10分 (0 = 不能安全完成)

身體動作評估	較差	適中	良好
**下肢功能**—5次坐到站	3		

評估下肢的功能性，以支持從坐姿到站姿以及回到坐姿的運動。 分數：1 – 10分 (0 = 不能安全完成)

自述身體健康狀況

過去六個月是否跌倒	否
快速起身時是否感到眩暈	是
室內燈光下視力情況	良好
每日服用處方類藥物的數量	<4

Copyright 2023, Booguu Co. Ltd. info@booguu.bio +852 3590 2685

名字	性別	年齡	身高	體重	日期

關於跌倒評估測試

肌力、平衡和協調性是滑倒或絆倒時重新控制住身體的關鍵。步行、平衡和“坐到站測試”評估了這些重要因素。在任何一項測試中，如果得分不超過3分，可能表明需要醫生去跟进随访。

可以考慮在日常活動中增加肌力、平衡和机动性的訓練，以提高或保持分數。在開始任何新的運動計劃之前，請務必先諮詢醫生。

步行表現

步行表現（步態）測試可測量下半身力量、協調性和穩定性。擁有健康步态的人對應起码的步行速度和前后一致的步伐。

步態分數較低的可能原因有速度慢、平拖步伐、有大且不必要的左右晃動，或前后不一致的步态。

站立平衡

平衡測試可測量身體保持站立時的微小動作。站立涉及三個系統：視覺、內耳（也稱為前庭系統）和本體感覺。當一個或多個系統發生改變時，平衡可能會受到影響。

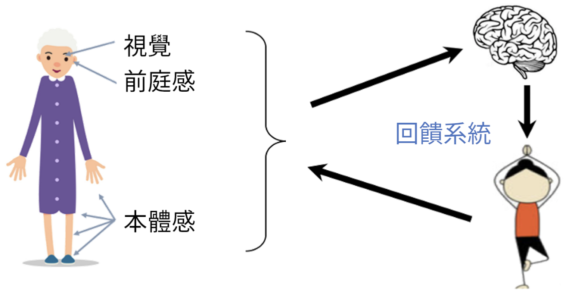

平衡得分低表示站立時身體經常進行大幅度的調整。若睜開眼睛和閉上眼睛的平衡存在明顯差異，說明高度依賴視覺來保持平衡，並且自我運動感覺系统可能存在問題。

“五次坐到站”測試

“坐到站測試”測量下半身力量和穩定性。健康的個體可以用穩定而充足的向上力量完成5個動作週期，並且所用時間是在年齡所對應的參考範圍內。

年齡	參考時間<br>(秒)
69以下	11.4
70 - 79	12.6
80以上	14.8

得分較低可能意味著下半身力量不足或身體動作不穩定。

Copyright 2023, Booguu Co. Ltd.   info@booguu.bio   +852 3590 2685

名字	性別	年齡	身高	體重	日期

步態評估（穿著鞋）

前進加速度

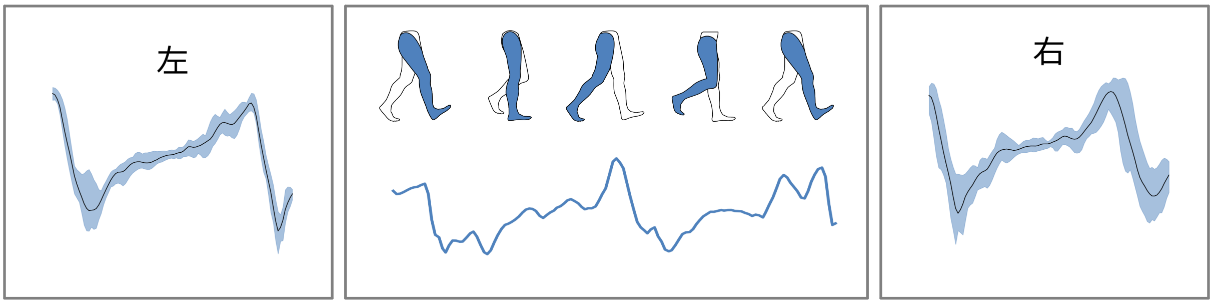

步態跌倒風險

7/10 適中

評估步行方式，包括時間和動作的變化性、強度和神經肌肉控制能力。

步態風險因素	分數	
步態表現		
每步能量	9	良好
上下運動幅度	7	適中
髖/骨盆穩定	8	良好
每步變化量	8	良好

更多資訊			參考範圍
步頻 (步/分)	113		> 96
時間非對稱性（%）	5.1		< 6.9
著陸力非對稱性（%）	1.4		< 14.5
平均著陸力 (x 體重)	1.45	1.47	1.44 - 2.05

Copyright 2023, Booguu Co. Ltd. info@booguu.bio +852 3590 2685

名字	性別	年齡	身高	體重	日期

平衡（靜態穩定性）評估

身體搖擺軌跡 & 長度

睜眼

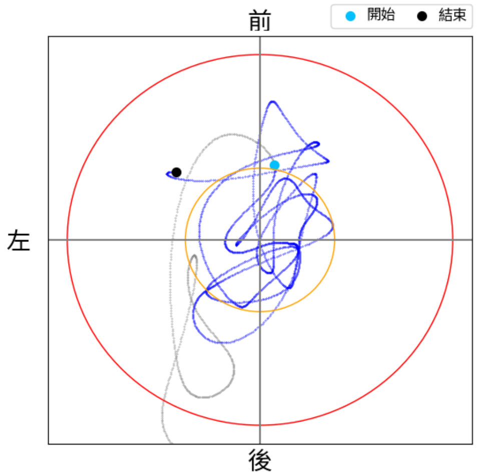

閉眼

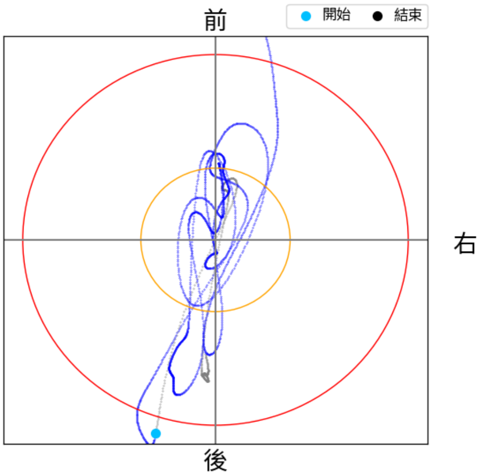

		參考	跌倒風險
睜眼搖擺長度	29.3cm	< 33.4cm	4/10
閉眼搖擺長度	29.0cm	< 38.0cm	6/10
阮柏氏測試			陰性

搖擺長度是身體移動路線的長度總和。搖擺長度越短，穩定性越好。

阮柏氏測試比較睜眼和閉眼的平衡結果。
阮柏氏測試結果為陽性可能表明需要依靠視覺來保持平衡，或者前庭和本體感受不足。

Copyright 2023, Booguu Co. Ltd. info@booguu.bio +852 3590 2685

名字	性別	年齡	身高	體重	日期

坐到站測試評估

5次坐到站

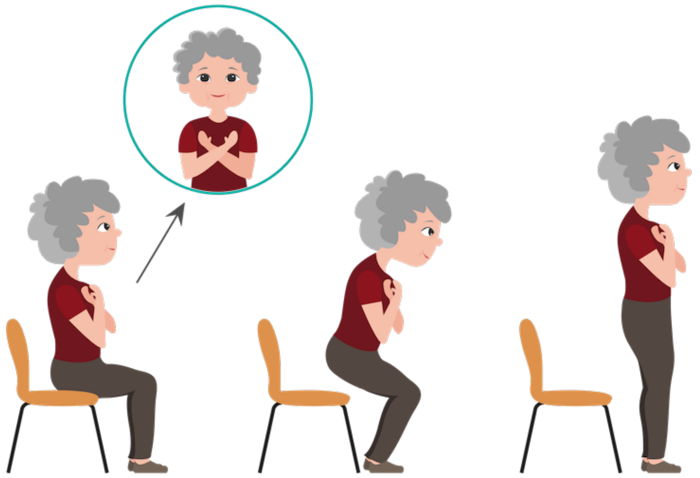

坐到站跌倒風險

3/10 較差

評估功能性下肢力量以及從坐姿過渡到站立的能力。

		參考
是否完成5次	否	
完成坐到站次數	4	
完成5次時間 (s)	不適用	< 11.40
到最後站起時間 (s)	12.27	
站起力量 (N)*	1.28	0.71 - 4.14
功能性效率	8	> 3
無法控制緩降	否	

* 乘以身體質量 (kg)

站起力量測量坐到站階段的垂直方向的加速度。力量越高，下肢肌肉功能越強。

功能性效率衡量從坐到站和從站到坐階段的動作轉換的質量。

無法控制緩降可能表示虛弱，疼痛，關節運動受限或註意力下降。

Copyright 2023, Booguu Co. Ltd. info@booguu.bio +852 3590 2685

aspire™

名字	性別	年齡	身高	體重	日期

提防環境陷阱：
https://www.elderly.gov.hk/tc_chi/healthy_ageing/home_safety/falls.html

做個精靈不倒翁：
https://www.elderly.gov.hk/tc_chi/books/files/fall_prevention/Fall_Prevention_booklet.pdf

對相關參數的說明，請參閱以下文件：
https://aspiremotion.com/rg.zh-hant.pdf

本報告所包含的信息屬於一般性質，並不代替您的醫生和其他醫療專業人員提供的建議。平衡與步態得分僅供參考。它不是醫學診斷或評估。這些陳述均不是治療任何特定疾病或與健康相關情況的建議。如果您懷疑自否己患有疾病或健康問題，應立即聯繫醫療保健專業人員。在開始任何平衡訓練、康復訓練、飲食協調、健康運動、服用補充劑或藥物治療計劃之前，請諮詢醫療保健專業人士。

Copyright 2023, Booguu Co. Ltd. info@booguu.bio +852 3590 2685
